# Financial protection of rural health insurance for patients with hypertension and diabetes: repeated cross-sectional surveys in rural China

**DOI:** 10.1186/s12913-016-1735-5

**Published:** 2016-09-08

**Authors:** Xiaoyun Liu, Xiaojie Sun, Yang Zhao, Qingyue Meng

**Affiliations:** 1 China Center for Health Development Studies, Peking University, Beijing, China; 2 Center for Health Management and Policy, Shandong University, Jinan, China

**Keywords:** New Cooperative Medical Scheme, Medical expenditure, Financial protection, Non-communicable chronic disease, China

## Abstract

**Background:**

The New Cooperative Medical Scheme (NCMS) in rural China has been expanding in both population coverage and benefit package. China has also established an essential medicine policy in recent years to further reduce patients’ medical expenditures and financial burden. This study aims to evaluate the impact of these policies on reducing medical expenditures and financial burden of patients diagnosed with hypertension and diabetes.

**Methods:**

This study used repeated cross-sectional surveys in 2011 and 2012 in three counties of Shandong Province. Outpatient and inpatient service expenditures and catastrophic health expenditures (CHE) were measured and analyzed.

**Results:**

Medical expenditures for outpatient services significantly increased for hypertensive and diabetic patients within a 1 year period, while inpatient service expenditures remained unchanged. Although NCMS increased its reimbursement rate, hypertensive and diabetic patients still heavily suffered CHE from both outpatient and inpatient services. Outpatient services were more important factors than inpatient services contributing to non-communicable chronic diseases (NCD) patients’ financial burden.

**Conclusions:**

The effects of NCMS expansion have been offset by the rapid escalation of medical expenditures. More attention should be paid to the design of NCMS benefit package to cover NCD outpatient services. There is also an urgent need to reform the current Fee for Service to other provider payment methods in order to control the escalating NCD medical expenditures.

**Electronic supplementary material:**

The online version of this article (doi:10.1186/s12913-016-1735-5) contains supplementary material, which is available to authorized users.

## Background

Since its establishment in 2003, the New Cooperative Medical Scheme (NCMS) in rural China has been expanding in both population coverage and benefit package [22]. By the end of 2012, the population coverage of NCMS had reached 98 %. The premium per person per year increased from 200 Yuan in 2011 to 250 Yuan in 2012. The Ministry of Health (MoH) reported that 55 % of inpatient medical expenditures and 49 % of outpatient medical expenditures were covered by NCMS in 2012 [11].

NCMS has increased the utilization of outpatient and inpatient health services [10, 23]. However, previous evidence in the early years of NCMS development showed no or only a modest impact on financial protection, due to low premiums and management challenges [13, 14, 24, 25]. With the rapid expansion in recent years, it is expected that NCMS will to some extent reduce the financial burden of rural residents’ medical expenses. In addition, China issued an essential medicine policy in 2011 for primary health care facilities. In this policy, doctors from rural township health centers can only prescribe from a list of 307 essential medical drugs, and they cannot make any profit from drug sales, while before the policy, they were allowed to charge a 15 % markup on prescription drugs. This essential medicine policy should have further reduced rural residents’ financial burden [5].

However, there is a “less clear storyline” regarding the real impact of these policies on medical expenditures and the financial burden [14]. On the one hand, many official statistics tend to use reimbursement rate (i.e. one minus copayment rate) to reflect the effect of NCMS policy. For example, MoH claimed that the reimbursement rate increased to 75 % in 2013 [11]. While reimbursement rate is an important indicator, it cannot really examine the actual financial burden, without sufficient attention being paid to out of pocket payments and patients’ capacity to pay. On the other hand, due to constraints in data availability, most studies analyzed NCMS’s impact on financial protection among all types of patients without proper risk adjustment of case mix. Wagstaff and colleagues [14] used household survey data to examine the out of pocket payments of all types of patients. They found NCMS did not reduce out-of-pocket expenses per outpatient visit or inpatient admission. Yip and Hsiao [21] also found that NCMS did not address medical impoverishment caused by expensive outpatient services for chronic conditions. Sun et al. [12] found that 15 % of families faced catastrophic health expenditures (CHE) due to health services for non-communicable chronic diseases (NCD) and NCMS did not play a significant role in reducing NCD patients’ financial burden. These two studies included patients with all types of chronic conditions. A different case mix between the samples may cause selection bias in estimating medical expenditures and financial burden [3].

The rapid rise of NCDs driven by urbanization, rising incomes, and aging poses major challenges for China’s health system. China had 177 million adults with hypertension in 2002. 22.8 % of Chinese people were overweight in 2002, and 7.1 % were obese [19]. NCDs contribute 68.6 % of the total disease burden in China [15]. Most NCD patients mainly use outpatient services rather than inpatient services for diagnosis and treatment, but NCMS, especially in the early years, mainly focused on inpatient services. NCD patients need to pay a very high copayment for outpatient services. A study using data from the early stage of NCMS development showed that NCD patients suffered a heavy financial burden because of outpatient services [12, 21]. As the rural health insurance scheme has rapidly developed in recent years, NCMS’s role in providing financial protection to rural residents may have greatly changed.

In this paper, we examine how the rapid expansions of NCMS bring financial protection to hypertensive and diabetic patients in three rural counties of Shandong Province. Our research hypothesis is that the expansion of NCMS benefit package together with the essential medicine policy since 2011 can reduce out of pocket payments and financial burden for rural hypertensive and diabetic patients.

## Methods

### Study settings

This study was conducted in three counties of Shandong Province (Pingyin, Liangshan, and Junan). They were selected to represent the overall socioeconomic development of Shandong Province (Table 1).

**Table 1:** General information about the NCMS development in the three counties

	Pingyin		Liangshan		Junan	
	2011	2012	2011	2012	2011	2012
NCMS Enrollment (%)	100	100	100	100	100	100
NCMS premium (Yuan)	250	250	250	300	150	300
Official copayment rate for outpatient service	70 %	70 %	70 % (with a ceiling of 100 Yuan)	70 % (with a ceiling of 100 Yuan)	70 % (with a ceiling of 120 Yuan)	70 % (with a ceiling of 170 Yuan)
Official copayment rate for inpatient service at township health center	30 %	15 %	35 %	30 %	35 %	15 %

We conducted repeated cross-sectional surveys with hypertensive and diabetic patients in the three counties. four out of seven townships from Pingyin, eight out of 14 townships from Liangshan, and eight out of 16 townships from Junan were randomly selected for the study. In each township, 20 hypertensive patients and 20 diabetic patients were selected from a NCD registration list using systematic random sampling methods. All patients were diagnosed at township health centers or higher level health facilities.

The first survey was conducted in June 2011. Master students from Shandong University and Peking University were trained to interview the patients. In total, 432 hypertensive patients and 388 diabetic patients were interviewed. The second cross-sectional survey was conducted in July 2012 in the same three counties and 20 townships, but with a new random sample of 414 hypertensive patients and 401 diabetic patients. Verbal informed consent for participation in the study was obtained from all participants.

A pre-decided questionnaire was applied in both surveys (Additional files 1 and 2). Patients’ demographic information (gender, age, education level, and household expenditures including food expenditures) were collected during the survey. Respondents were asked to self-report information on medical expenditures and NCMS reimbursement. Questions about outpatient expenditures in the previous month and inpatient expenditures in the previous year were asked in order to reduce recall bias.

In data analysis, comparisons of participants’ age, gender, marriage status, education level, household expenditures and health insurance status were made between the two samples in 2011 and 2012, in order to examine the comparability of the two groups. Medical expenditures for outpatient and inpatient services, NCMS copayment, and incidence of CHE were analyzed. Median and quartile intervals were applied to describe medical expenditures whose distribution was positively skewed. A significance test between the 2 years was made using Chi Square test for categorical data, t test for normal distribution continuous data, and rank sum test for non-normal distribution data. CHE was defined when patients’ medical expenditures were above 40 % of their annual household nonfood expenditures [17].

To understand the change of outpatient and inpatient expenditures between 2011 and 2012, and to analyze the potential influence of the increasing NCMS premium level on medical expenditures, we did linear regression analysis. The dependent variables were monthly outpatient expenditures and annual inpatient expenditures. The main independent variables included the NCMS premium level and year (2011 and 2012), while controlling NCD patients’ sex, age and education (illiterate or non-illiterate) as the confounding factors. Stata 12.0 was used for data analysis.

## Results

The two samples were highly homogeneous in terms of their demographic indicators. All statistical comparisons between the two samples in Table 2 were insignificant at 5 % level. The sampled hypertensive and diabetic patients were mostly old people, with an average age older than 60 years. About one-third of hypertensive patients and one-fourth of diabetic patients were male. More than 80 % of the participants were married. They had received a very limited education. About two-thirds were illiterate and very few received a college education. These NCD patients were rather poor rural residents, and their average household expenditures (including food, medical services, and other expenditures) were only 7000–9000 Yuan per year.

**Table 2:** General information on hypertensive and diabetic patients in three rural counties in Shandong Province

	Hypertension		Diabetes	
	2011	2012	2011	2012
Sample size (*n*)	432	414	388	401
Age (mean, SD)	64.7 (10.1)	64.3 (10.4)	61.2 (9.4)	62.3 (9.9)
Sex (% of male)	36.6	34.1	24.5	26.9
Marriage (% of married)	80.1	82.9	86.3	82.0
Education (%)				
Illiterate	64.8	65.2	63.7	65.8
Primary school	19.7	18.1	18.6	20.7
High school	15.5	16.7	17.5	13.2
College and above	0	0	0.3	0.3
Annual household expenditure Yuan (Median, P25 P75)	7000 (4000–10950)	7200 (3500–12000)	9000 (5500–14550)	8800 (5100–13600)
NCMS coverage (%)	96.5	98.1	97.4	97.5

*P* > 0.05 for all comparisons between 2011 and 2012

Table 3 shows that, for hypertensive patients, the number of outpatient visits in the last month increased from 1.8 in 2011 to 2.2 in 2012 (*p* < 0.01). The median medical expenditures for outpatient service increased from 20 Yuan to 40 Yuan per month. Although the copayment rate was reduced from 83.2 to 70.2 %, the proportion of hypertensive patients suffering from CHE due to annual expenditures for outpatient services increased from 11.8 to 23.0 % (*p* < 0.05).

**Table 3:** Outpatient service utilization and expenditure for hypertensive and diabetic patients in 2011 and 2012

	Hypertension		Diabetes	
	2011	2012	2011	2012
No. of OP visits in the last month (Mean)	1.8	2.2**	1.6	1.7
Monthly outpatient expenditure (Yuan)				
Mean (SD)	56.6 (226.8)	140.6 (481.8)**	123.5 (147.8)	252.9 (618.7)**
Median (P25 P75)	20 (10–50)	40 (15–110)**	73.5 (30–157.5)	100 (30–260)*
% of copayment	83.2	70.2**	85.7 %	78.2 %**
CHE incidence (%)	11.8	23.0**	22.1	37.5**

*: 0.01 < *P* < 0.05; **: *P* < 0.01

For diabetic patients, the number of outpatient visits had a slight increase (from 1.6 to 1.7, *P* = 0.10). Monthly expenditures for diabetics services increased from 73.5 Yuan in 2011 to 100 Yuan in 2012 (*p* < 0.05). As a result of increasing annual expenditures for outpatient services, the proportion of CHE increased from 22.1 to 37.5 % in a 1 year period, despite the decreasing copayment level (from 85.7 to 75.2 %).

Table 4 shows the regression analysis results of monthly outpatient expenditures among those who used outpatient services. After controlling for confounding factors, the monthly outpatient medical expenditures still had a 98.5 Yuan increase for hypertensive patients and a 135.1 Yuan increase for diabetic patients (*P* < 0.01) in the 1 year period (2011–2012). The number of outpatient visits in the last month had a positive association with the monthly outpatient medical expenditures. The increase of NCMS premium level did not show any significant association with outpatient expenditures.

**Table 4:** Regression analysis of monthly outpatient expenditure for hypertensive and diabetic patients in 2011 and 2012

	Hypertension	Diabetes
	β (95 % CI)	β (95 % CI)
Constant	111.1 (−138.9, 361.1)	12.4 (−307.8, 332.6)
Sex		
Male (Reference)	0	0
Female	−1.4 (−60.0, 57.2)	40.2 (−37.9, 118.3)
Age	−1.0 (−3.6, 1.7)	−0.9 (−4.3, 2.6)
Education level		
Non-illiterate (Reference)	0	0
Illiterate	55.0 (−7.4, 117.5)	58.8 (−17.6, 135.2)
No. of outpatient visits	24.1 (10.9, 37.3)**	64.3 (44.9, 83.8)**
NCMS premium	−0.3 (−1.0, 0.3)	−0.2 (−1.0, 0.6)
Year		
2011 (Reference)	0	0
2012	98.5 (25.6, 171.3)**	135.1 (44.0, 226.2)**
Adjusted R-squared	0.026**	0.071**

*: 0.01 < *P* < 0.05; **: *P* < 0.01

The inpatient service utilization rate for hypertensive patients slightly increased from 16.0 % in 2011 to 16.7 % in 2012 (*P* > 0.05). The median health expenditures per inpatient admission remained at 4000 Yuan in the 2 years, but the mean value increased from 4516.6 Yuan to 6734.2 Yuan (*P* > 0.05). The NCMS copayment rate decreased from 62.8 to 57.8 % (*P* > 0.05). The rate of CHE due to hypertensive inpatient services was 42.0 % in 2011 and 43.5 % in 2012, with no significant difference (Table 5).

**Table 5:** Inpatient expenditures and NCMS reimbursement for hypertensive and diabetic patients in 2011 and 2012

	Hypertension		Diabetes	
	2011	2012	2011	2012
% of patients using inpatient services	16.0	16.7	19.3	21.7
Inpatient expenditures				
Mean (SD)	4516.6 (4771.5)	6734.2 (10734.8)	5023.5 (4559.1)	4549.3 (3386.0)
Median (P25 P75)	4000 (1680–6000)	4000 (2000–6734)	4000 (2000–6000)	4000 (3000–6000)
% of copayment for inpatient services	62.8	57.8	66.0	50.4**
CHE incidence (%)	42.0	43.5	36.0	32.2

*: 0.01 < *P* < 0.05; **: *P* < 0.01

For diabetic patients, median expenditures for inpatient services also remained unchanged in the 2 years. The copayment rate was reduced from 66.0 to 50.4 % (*P* < 0.01). The rate of CHE due to inpatient services slightly decreased from 36.0 to 32.2 % (*P* > 0.05).

Table 6 shows the regression analysis results of inpatient expenditures among those who used inpatient services. The inpatient expenditures for hypertensive and diabetic patients did not significantly increase over the 1 year period (*P* > 0.05). The increase of the NCMS premium level also did not increase the inpatient expenditures (*P* > 0.05).

**Table 6:** Regression analysis of annual inpatient expenditures for hypertensive and diabetic patients in 2011 and 2012

	Hypertension	Diabetes
	β (95 % CI)	β (95 % CI)
Constant	8508.9 (−5438.1, 22455.8)	5043.1 (−1708.6, 11794.9)
Sex		
Male (Reference)	0	0
Female	−3396.7 (−6779.5, −13.8)*	216.3 (−1299.4, 1732.0)
Age	−47.4 (−197.1, 102.3)	−10.8 (−79.2, 57.7)
Education level		
Non-illiterate (Reference)	0	0
Illiterate	−1664.7 (−5359.6, 2030.2)	−1208.6 (−2633.1, 215.8)
NCMS premium	27.0 (−11.1, 65.0)	5.2 (−12.5, 22.9)
Year		
2011 (Reference)	0	0
2012	245.1 (−3619.6, 4109.7)	−889.4 (−2739.2, 960.4)
Adjusted R-squared	0.057*	0.006

*: 0.01 < *P* < 0.05; **: *P* < 0.01

It should be noted that the adjusted R-squared are less than 10 % in all regression analyses (Tables 4 and 6), which implies that there should be other factors than included in the models influencing the changes of outpatient and inpatient expenditures.

## Discussion

NCDs are major sources of disease burden in China, and will keep on increasing due to rapid population aging. NCDs can bring great financial loss and economic burden to patients and their family [15]. This study aimed to analyze the potential impact of NCMS expansions on NCD patients’ medical expenditures and their financial burden.

### Increase of outpatient service utilization and medical expenditures

The study found that within a 1 year period, utilization and expenditures of outpatient services significantly increased for hypertensive and diabetic patients. Utilization and expenditure of inpatient services remained unchanged. Hypertension and diabetes are prevalent among the elderly population in rural China [9]. Old people are usually poor and do not have a regular income. A small but regular expense on hypertensive and diabetic outpatient service could be catastrophic to them, and prevent them using health services [8, 16]. Therefore, reduction of the copayment rate may release NCD patients’ unmet need and be a stimulus for them to use more outpatient services.

In addition, along with the health system reform, China also implemented an essential public health service package which is equally provided to all citizens. Disease management for NCD patients is included in the package [20]. The public health services may update patients’ knowledge on NCD and change their perception, and therefore encourage people to use more outpatient services.

More outpatient visits inevitably increased patients’ medical expenditures. The reason why outpatient expenditures had a large increase is possibly related to the implementation of the essential medicine policy. Under this policy, some anti-hypertensive and anti-diabetic drugs were no longer available in the township health centers. Patients had to turn to high level hospitals or private drug stores to purchase their drugs, which may have resulted in higher medical expenses. This phenomenon of shortage of drug availability in township health centers was also reported elsewhere [7].

There are multifold reasons for the rapid increase of medical expenditures. Rising prices of health inputs and introduction of more expensive technologies or drugs are among the main driving forces [1, 4]. The behavior of health providers in revenue generation can also contribute to the increasing medical expenses. Fee for service is still the main provider payment method in China’s health system. It has been frequently reported that this payment method has led to distorted motivation for health workers to over supply medical services. This in turn drives the escalation of medical expenditures [2, 6].

### Medical expenditure place a heavy financial burden on hypertensive and diabetic patients

Medical expenditures of both outpatient and inpatient services place a heavy financial burden on hypertensive and diabetic patients. This is especially so for outpatient services, because almost all hypertensive and diabetic patients had outpatient services while only 16 % of hypertensive patients and 20 % of diabetic patients had inpatient services. Considering all hypertensive patients (rather than only those who used health services), outpatient service caused 11.2 % CHE in 2011 and 21.7 % CHE in 2012. This is much higher than the incidence of CHE caused by inpatient services (6.7 % in 2011 and 7.3 % in 2012). The same conclusion can be drawn for diabetic patients. Therefore outpatient services are more important factors contributing to NCD patients’ financial burden. The importance of outpatient services for NCD patients was also reported by other authors [12, 21].

### Role of NCMS in providing financial protection to hypertensive and diabetic patients

The primary goal of NCMS is to prevent rural residents from falling into poverty trap due to medical expenditures. Previous studies showed that NCMS had a modest impact on controlling medical expenditure and reducing patients’ financial burden [14, 25]. In this study, regression analysis found that the increase of NCMS premium had no significant impact in reducing NCD patients’ medical expenditures. NCD patients’ financial burden kept on increasing regardless of the expansions in NCMS coverage and benefit package. The main explanation is that the speed of medical expenditures increase is faster than the speed of NCMS expansions, and therefore compromises the effect of NCMS on financial protection.

### Limitation of this study

The study used repeated cross-sectional surveys to analyze the changes of medical expenditures and the financial burden of NCD patients. The main policy changes between the two surveys included the increase of NCMS premium and the essential medicine policy. Along with these changes, there were some other health system reform measures such as the essential public health services package. The design of this study cannot fully distinguish the effect of these different policy changes.

The study was only conducted in one province of China. Due to the vast variation of the health system across China, the research findings may not be generalized to other settings without caution.

A large discrepancy between the official copayment rate and real copayment rate exists in China’s rural health insurance scheme. This is mainly because some medical services (including drugs) are not included in NCMS’s benefit packages. While the official copayment rate only considers the eligible services covered by the benefit package, the real reimbursement rate uses the overall medical expenditures for the calculation.

### Policy implications

This study shows that hypertensive and diabetic patients suffer heavily from CHE, especially due to outpatient services. As NCMS has nearly reached universal population coverage and has also expanded its benefit package, more attention should be paid to NCDs such as hypertension and diabetes in the design of the benefit package. The copayment level for outpatient services should be significantly reduced in order to reduce the financial burden of the elderly and poor NCD patients in rural China.

There is an urgent need to reform the current Fee for Service to other provider payment methods in order to control escalating medical expenditures. This urgent need has not yet been widely recognized in the context of NCMS in China, especially among the NCMS fund managers at the local level. The NCMS premium level has been increasing every year, from 30 Yuan in 2003 to 250 Yuan in 2012. The local NCMS managers are kept busy adjusting the benefit package design to use the fund wisely according to the national guideline [18]. They have not yet shifted their priority to cost control measures. As the rural health insurance system begins to mature after 10 years implementation, provider payment reform should come to the policy agenda in order for NCMS to better provide financial protection to the rural population.

## Conclusion

The effects of NCMS expansion have been offset by the rapid escalation of medical expenditures. More attentions should be paid to the design of NCMS benefit package to cover NCD outpatient services. There is also an urgent need to reform the current Fee for Service to other provider payment methods in order to control the escalating NCD medical expenditures.

## Additional files

Additional file 1: Survey questionnaire for hypertension patients. (DOCX 21 kb) Additional file 2: Survey questionnaire for diabetes patients. (DOCX 21 kb)

## Abbreviations

CHE: Catastrophic health expenditure
CI: Confidence interval
MoH: Ministry of Health
NCD: Non-communicable chronic disease
NCMS: New Cooperative Medical Scheme
OP: Outpatient
SD: Standard deviation
