# Supplementary material for: Financial protection of rural health insurance for patients with hypertension and diabetes: repeated cross-sectional surveys in rural China
Source: BMC Health Serv Res. 2016 Sep 8;16(1):481. doi: 10.1186/s12913-016-1735-5 (PMC5017002; doi:10.1186/s12913-016-1735-5)
Supplement: Additional file 2: — Survey questionnaire for diabetes patients. (DOCX 21 kb) [file 12913_2016_1735_MOESM2_ESM.docx]

**Supplementary file 2:**

**Survey Questionnaire for Diabetes Patients**

Good morning/afternoon. We are a research team from Shandong University and Peking University to study the provider payment methods for the New Medical Cooperative Scheme (NCMS). We would like to talk about your health and health care. The interview will take about 10 minutes of your time. Your participation is voluntary. The information you provide will be kept confidential. Your participation in the survey will not affect your future health services in any way. Should you have any questions or queries about the survey, please let us know.

**Part 1: General information about the interview**

|  | Code |
| --- | --- |
| 1. No of interviewee: |  |
| 1. Location of interview: county township village |  |
| 1. Date and time of interview: year month date time |  |
| 1. Name of enumerator: |  |
| 1. Name of quality inspector: |  |

**Part two: Information about disease, health care and cost**

| 1. Time of diagnosis with Diabetes： year month day |  |
| --- | --- |
| 1. Where was the diagnosis made?   1. Township health center; 2. County hospital; 3. Higher hospital; 4. Other health facilities |  |
| 1. Type of Diabetes: 1. Type one; 2. Type two; 3. DK |  |
| 1. Do you have any complication of diabetes? 1.yes; 2.no(jump to 5) |  |
| If yes, the first complication |  |
| Second |  |
| Third |  |
| 1. How many time did you visit a doctors or a pharmacist due to your Diabetes in the last month? |  |
| Inc. private clinic |  |
| Village clinic |  |
| Township health center |  |
| County hospital |  |
| Drug store |  |
| Other health facilities |  |
| 1. Do you take any medicine for diabetes now? 1. yes; 2. No (jump to 9) |  |
| 1. What medicine? 1. ; 2. DK |  |
| 1. Way of administration? 1. ; 2. DK |  |
| 1. How much do you spend on diabetes every month? Yuan (Exc hospitalization cost) |  |
| Including registration fee Yuan |  |
| Test and examination fee Yuan |  |
| Drug cost Yuan (including those in drug store) |  |
| How much can NCMS reimburse? Yuan |  |
| How much can other insurance reimburse? Yuan |  |
| 1. Did you have hospitalization for diabetes in the last year?   1. yes, times; 2. No (jump to part 3) |  |
| 1. Where was your most recent hospitalization episode in the last year? 1. Township health center; 2. County hospital; 3. Higher hospital; 4. Other health facilities |  |
| 1. How much did you spend for this hospitalization (direct medical cost) Yuan |  |
| 1. How much can NCMS reimburse? Yuan |  |
| 1. How much can other insurance reimburse? Yuan |  |

**Part Three: Disease management**

| 1. Do you take medicine regularly following doctors’ advice? 1. Yes (jump to 3); 2. No |  |
| --- | --- |
| 1. Why can’t you take medicine regularly following doctors’ advice?：1. financial difficulty; 2. Symptom relief, not need to take more medicine; 3. Ineffective medicine; 4. Severe adverse effect; 5. No time or forget to take medicine; 6. other |  |
| 1. After diagnosis with Diabetes, how often do you have blood sugar test? 1. Once every days; 2. never; 3. DK |  |
| 1. After diagnosis with Diabetes, how often do you have a follow up visit to a doctor? 1. Once every days; 2. never; 3. DK |  |
| 1. Do you stop smoking after diagnosis with Diabetes? 1. Did not smoke before; 2. Fully stop smoking; 3. Reduce smoking; 4. No change at all |  |
| 1. Do you stop alcohol drinking after diagnosis with Diabetes? 1. Did not drink alcohol before; 2. Fully stop alcohol drinking; 3. Reduce alcohol drinking; 4. No change at all |  |
| 1. Do you have diet control after diagnosis with Diabetes? 1. Yes, 2. Only partly; 3. No change at all |  |
| 1. Do you have regular physical excise after diagnosis with Diabetes? 1. Yes; 2. Occasionally; 3. No change at all |  |
| 1. Do you have a follow-up visit from any health workers after diagnosis with Diabetes? 1. yes; 2. No (jump to part 4) |  |
| 1. Who do the follow-up visit? 1. Village clinic; 2. THC doctor; 3. Other |  |
| 1. How do they visit you? 1. By telephone; 2. Home visit; 3. Ask me to their health facility; 4. other |  |
| 1. Does the follow-up doctor have your medical file 1. yes; 2. no; 3. DK |  |

**Part four: interviewee’s general information**

| 1. Gender : 1. male; 2. female |  |
| --- | --- |
| 1. Date of Birth: ( year/ month) |  |
| 1. Ethnic group: 1. Han; 2. Hui; 3. Man; 4. Other |  |
| 1. Marriage status: 1.single; 2.married; 3.divorced; 4.Widowed. |  |
| 1. Education : 1.illiterate; 2.primary school; 3.junior middle school; 4.senior middle school or vocational school; 5.college and above |  |
| 1. Occupation: 1.farming; 2.household; 3.unemployed; 4.managerial or professional job; 5.migrant worker; 6.self employed; 7.other |  |
| 1. Household income in the last year Yuan |  |
| Inc. salary income Yuan |  |
| Migrant workers’ income Yuan |  |
| Agriculture income Yuan |  |
| Other income Yuan |  |
| 1. Household expenditure Yuan |  |
| Inc. food expenditure Yuan |  |
| Educational expenditure Yuan |  |
| Medical expenditure Yuan |  |
| Other expenditure Yuan |  |
| 1. What health insurance scheme do you have now? (multiple choice): 1.None; 2.NCMS; 3.urban employment based basic health insurance; 4.urban resident medical health insurance; 5.private insurance; 6.other |  |
